# Fixpoint Theory – Upside Down

**DOI:** 10.1007/978-3-030-71995-1_4

**Published:** 2021-03-23

**Authors:** Paolo Baldan, Richard Eggert, Barbara König, Tommaso Padoan

**Affiliations:** 8 University of Oxford, Oxford, UK; 9 Sorbonne Université - LIP6, Paris, France; 10 Università di Padova, Padova, Italy; 11 Universität Duisburg-Essen, Duisburg, Germany

## Abstract

Knaster-Tarski’s theorem, characterising the greatest fix- point of a monotone function over a complete lattice as the largest post-fixpoint, naturally leads to the so-called coinduction proof principle for showing that some element is below the greatest fixpoint (e.g., for providing bisimilarity witnesses). The dual principle, used for showing that an element is above the least fixpoint, is related to inductive invariants. In this paper we provide proof rules which are similar in spirit but for showing that an element is above the greatest fixpoint or, dually, below the least fixpoint. The theory is developed for non-expansive monotone functions on suitable lattices of the form $\mathbb {M}^Y$, where *Y* is a finite set and $\mathbb {M}$ an MV-algebra, and it is based on the construction of (finitary) approximations of the original functions. We show that our theory applies to a wide range of examples, including termination probabilities, behavioural distances for probabilistic automata and bisimilarity. Moreover it allows us to determine original algorithms for solving simple stochastic games.

### 1 Introduction

Fixpoints are ubiquitous in computer science as they allow to provide a meaning to inductive and coinductive definitions (see, e.g., [26,23]). A monotone function $f : L \to L$ over a complete lattice $(L, \sqsubseteq)$, by Knaster-Tarski's theorem [28], admits a least fixpoint $\mu f$ and greatest fixpoint $\nu f$ which are characterised as the least pre-fixpoint and the greatest post-fixpoint, respectively. This immediately gives well-known proof principles for showing that a lattice element $l \in L$ is *below* $\nu f$ or *above* $\mu f$

$$\frac{l \sqsubseteq f(l)}{l \sqsubseteq \nu f} \qquad\qquad \frac{f(l) \sqsubseteq l}{\mu f \sqsubseteq l}$$

On the other hand, showing that a given element $l$ is *above* $\nu f$ or *below* $\mu f$ is more difficult. One can think of using the characterisation of least and largest fixpoints via Kleene's iteration. E.g., the largest fixpoint is the least element of the (possibly transfinite) descending chain obtained by iterating $f$ from $\top$. Then showing that $f^i(\top) \sqsubseteq l$ for some $i$, one concludes that $\nu f \sqsubseteq l$. This proof principle is related to the notion of ranking functions. However, this is a less satisfying notion of witness since $f$ has to be applied $i$ times, and this can be inefficient or unfeasible when $i$ is an infinite ordinal.

The aim of this paper is to present an alternative proof rule for this purpose for functions over lattices of the form $L = \mathbb{M}^Y$ where $Y$ is a finite set and $\mathbb{M}$ is an MV-chain, i.e., a totally ordered complete lattice endowed with suitable operations of sum and complement. This allows us to capture several examples, ranging from ordinary relations, for dealing with bisimilarity, behavioural metrics, termination probabilities and simple stochastic games.

Assume $f : \mathbb{M}^Y \to \mathbb{M}^Y$ monotone and consider the question of proving that some fixpoint $a : Y \to \mathbb{M}$ is the largest fixpoint $\nu f$. The idea is to show that there is no "slack" or "wiggle room" in the fixpoint $a$ that would allow us to further increase it. This is done by associating with every $a : Y \to \mathbb{M}$ a function $f_a^\#$ on $\mathbf{2}^Y$ whose greatest fixpoint gives us the elements of $Y$ where we have a potential for increasing $a$ by adding a constant. If no such potential exists, i.e. $\nu f_a^\#$ is empty, we conclude that $a$ is $\nu f$. A similar function $f_\#^a$ (specifying decrease instead of increase) exists for the case of least fixpoints. Note that the premise is $\nu f_\#^a = \emptyset$, i.e. the witness remains coinductive. The proof rules are:

$$\frac{f(a) = a \qquad \nu f_a^\# = \emptyset}{\nu f = a} \qquad \frac{f(a) = a \qquad \nu f_\#^a = \emptyset}{\mu f = a}$$

For applying the rule we compute a greatest fixpoint on $\mathbf{2}^Y$, which is finite, instead of working on the potentially infinite $\mathbb{M}^Y$. The rule does not work for all monotone functions $f : \mathbb{M}^Y \to \mathbb{M}^Y$, but we show that whenever $f$ is non-expansive the rule is valid. Actually, it is not only sound, but also reversible, i.e., if $a = \nu f$ then $\nu f_a^\# = \emptyset$, providing an if-and-only-if characterisation.

Quite interestingly, under the same assumptions on $f$, using a restricted function $f_a^*$, the rule can be used, more generally, when $a$ is just a *pre-fixpoint* ($f(a) \sqsubseteq a$) and it allows to conclude that $\nu f \sqsubseteq a$. A dual result holds for *post-fixpoints* in the case of least fixpoints.

$$\frac{f(a) \sqsubseteq a \qquad \nu f_a^* = \emptyset}{\nu f \sqsubseteq a} \qquad \frac{a \sqsubseteq f(a) \qquad \nu f_*^a = \emptyset}{a \sqsubseteq \mu f}$$

As already mentioned, the theory above applies to many interesting scenarios: witnesses for non-bisimilarity, algorithms for simple stochastic games [11] and lower bounds for termination probabilities and behavioural metrics in the setting of probabilistic systems [1] and probabilistic automata [2]. In particular we were inspired by, and generalise, the self-closed relations of Fu [16], also used in [2].

*Motivating Example.* Consider a Markov chain $(S, T, \eta)$ with a finite set of states $S$, where $T \subseteq S$ are the terminal states and every state $s \in S \backslash T$ is associated with a probability distribution $\eta(s) \in \mathcal{D}(S)$.[3] Intuitively, $\eta(s)(s')$ denotes the probability of state $s$ choosing $s'$ as its successor. Assume that, given a fixed state $s \in S$, we want to determine the termination probability of $s$, i.e. the probability of reaching any terminal state from $s$. As a concrete example, take the Markov chain given in Fig. 1, where $u$ is the only terminal state.

[3] $\mathcal{D}(S)$ is the set of all maps $p : S \to [0,1]$ such that $\sum_{s \in S} p(s) = 1$.

$$\mathcal{T} : [0,1]^S \to [0,1]^S$$

$$\mathcal{T}(t)(s) = \begin{cases} 1 & \text{if } v \in T \\ \sum_{s' \in S} \eta(s)(s') \cdot t(s') & \text{otherwise} \end{cases}$$

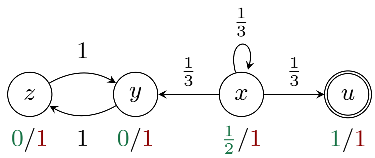

Fig. 1: Function $\mathcal{T}$ (left) and a Markov chain with two fixpoints of $\mathcal{T}$ (right)

The termination probability arises as the least fixpoint of a function $\mathcal{T}$ defined as in Fig. 1. The values of $\mu\mathcal{T}$ are indicated in green (left value).

Now consider the function $t$ assigning to each state the termination probability written in red (right value). It is not difficult to see that $t$ is another fixpoint of $\mathcal{T}$, in which states $y$ and $z$ convince each other incorrectly that they terminate with probability 1, resulting in a vicious cycle that gives "wrong" results. We want to show that $\mu\mathcal{T} \neq t$ without knowing $\mu\mathcal{T}$. Our idea is to compute the set of states that still has some "wiggle room", i.e., those states which could reduce their termination probability by $\delta$ if all their successors did the same. This definition has a coinductive flavour and it can be computed as a greatest fixpoint on the finite powerset $\mathbf{2}^S$ of states, instead of on the infinite lattice $S^{[0,1]}$.

We hence consider a function $\mathcal{T}_{\#}^{t} : \mathbf{2}^{[S]^t} \to \mathbf{2}^{[S]^t}$, dependent on $t$, defined as follows. Let $[S]^t$ be the set of all states $s$ where $t(s) > 0$, i.e., a reduction is in principle possible. Then a state $s \in [S]^t$ is in $\mathcal{T}_{\#}^{t}(S')$ iff $s \notin T$ and for all $s'$ for which $\eta(s)(s') > 0$ it holds that $s' \in S'$, i.e. all successors of $s$ are in $S'$.

The greatest fixpoint of $\mathcal{T}_{\#}^{t}$ is $\{y, z\}$. The fact that it is not empty means that there is some "wiggle room", i.e., the value of $t$ can be reduced on the elements $\{y, z\}$ and thus $t$ cannot be the least fixpoint of $f$. Moreover, the intuition that $t$ can be improved on $\{y, z\}$ can be made precise, leading to the possibility of performing the improvement and search for the least fixpoint from there.

*Contributions.* In the paper we formalise the theory outlined above, showing that the proof rules work for non-expansive monotone functions $f$ on lattices of the form $\mathbb{M}^Y$, where $Y$ is a finite set and $\mathbb{M}$ an MV-algebra (§3 and §4). Additionally, given a decomposition of $f$ we show how to obtain the corresponding approximation compositionally (§5). Then, in order to show that our approach covers a wide range of examples and allows us to derive original algorithms, we discuss various applications: termination probability, behavioural distances for probabilistic automata and bisimilarity (§6) and simple stochastic games (§7).

Proofs and further material can be found in the full version of the paper [5].

### 2 Lattices and MV-Algebras

In this section, we review some basic notions used in the paper.

A preordered or partially ordered set $(P, \sqsubseteq)$ is often denoted simply as $P$, omitting the order relation. Given $x, y \in P$, with $x \sqsubseteq y$, we denote by $[x, y]$ the interval $\{z \in P \mid x \sqsubseteq z \sqsubseteq y\}$. The *join* and the *meet* of a subset $X \subseteq P$ (if they exist) are denoted $\bigsqcup X$ and $\bigsqcap X$, respectively.

A *complete lattice* is a partially ordered set $(L, \sqsubseteq)$ such that each subset $X \subseteq L$ admits a join $\bigsqcup X$ and a meet $\bigsqcap X$. A complete lattice $(L, \sqsubseteq)$ always has a least element $\bot = \bigsqcup \emptyset$ and a greatest element $\top = \bigsqcap \emptyset$.

A function $f : L \to L$ is *monotone* if for all $l, l' \in L$, if $l \sqsubseteq l'$ then $f(l) \sqsubseteq f(l')$. By Knaster-Tarski's theorem [28, Thm. 1], any monotone function on a complete lattice has a least and a greatest fixpoint, denoted respectively $\mu f$ and $\nu f$, characterised as the meet of all pre-fixpoints respectively the join of all post-fixpoints: $\mu f = \bigsqcap\{l \mid f(l) \sqsubseteq l\}$ and $\nu f = \bigsqcup\{l \mid l \sqsubseteq f(l)\}$.

Let $(C, \sqsubseteq)$, $(A, \leq)$ be complete lattices. A *Galois connection* is a pair of monotone functions $\langle \alpha, \gamma \rangle$ such that $\alpha : C \to A$, $\gamma : A \to C$ and for all $a \in A$ and $c \in C$: $\alpha(c) \leq a \iff c \sqsubseteq \gamma(a)$. Equivalently, for all $a \in A$ and $c \in C$, (i) $c \sqsubseteq \gamma(\alpha(c))$ and (ii) $\alpha(\gamma(a)) \leq a$. In this case we will write $\langle \alpha, \gamma \rangle : C \to A$. For a Galois connection $\langle \alpha, \gamma \rangle : C \to A$, the function $\alpha$ is called the left (or lower) adjoint and $\gamma$ the right (or upper) adjoint.

Galois connections are at the heart of abstract interpretation [13,14]. In particular, when $\langle \alpha, \gamma \rangle$ is a Galois connection, given $f^C : C \to C$ and $f^A : A \to A$, monotone functions, if $f^C \circ \gamma \sqsubseteq \gamma \circ f^A$, then $\nu f^C \sqsubseteq \gamma(\nu f^A)$. If equality holds, i.e., $f^C \circ \gamma = \gamma \circ f^A$, then greatest fixpoints are preserved along the connection, i.e., $\nu f^C = \gamma(\nu f^A)$.

Given a set $Y$ and a complete lattice $L$, the set of functions $L^Y = \{f \mid f : Y \to L\}$, endowed with pointwise order, i.e., for $a, b \in L^Y$, $a \sqsubseteq b$ if $a(y) \sqsubseteq b(y)$ for all $y \in Y$, is a complete lattice.

In the paper we will mostly work with lattices of the kind $\mathbb{M}^Y$ where $\mathbb{M}$ is a special kind of lattice with a rich algebraic structure, i.e. an MV-algebra [21].

**Definition 1 (MV-algebra).** *An* MV-algebra *is a tuple* $\mathbb{M} = (M, \oplus, 0, \overline{(\cdot)})$ *where* $(M, \oplus, 0)$ *is a commutative monoid and* $\overline{(\cdot)} : M \to M$ *maps each element to its* complement, *such that for all* $x, y \in M$ *(1)* $\overline{\overline{x}} = x$; *(2)* $x \oplus \overline{0} = \overline{0}$; *(3)* $\overline{(\overline{x} \oplus y)} \oplus y = \overline{(\overline{y} \oplus x)} \oplus x$.

*We denote* $1 = \overline{0}$, *multiplication* $x \otimes y = \overline{\overline{x} \oplus \overline{y}}$ *and subtraction* $x \ominus y = x \otimes \overline{y}$.

**Definition 2 (natural order).** *Let* $\mathbb{M} = (M, \oplus, 0, \overline{(\cdot)})$ *be an MV-algebra. The* natural order *on* $\mathbb{M}$ *is defined, for* $x, y \in M$, *by* $x \sqsubseteq y$ *if* $x \oplus z = y$ *for some* $z \in M$. *When* $\sqsubseteq$ *is total* $\mathbb{M}$ *is called an* MV-chain.

The natural order gives an MV-algebra a lattice structure where $\bot = 0$, $\top = 1$, $x \sqcup y = (x \ominus y) \oplus y$ and $x \sqcap y = \overline{\overline{x} \sqcup \overline{y}} = x \otimes (\overline{x} \oplus y)$. We call the MV-algebra *complete*, if it is a complete lattice, which is not true in general, e.g., $([0,1] \cap \mathbb{Q}, \leq)$.

*Example 3.* A prototypical example of an MV-algebra is $([0,1], \oplus, 0, \overline{(\cdot)})$ where $x \oplus y = \min\{x + y, 1\}$ and $\overline{x} = 1 - x$ for $x, y \in [0,1]$. This means that $x \otimes y = \max\{x + y - 1, 0\}$ and $x \ominus y = \max\{0, x - y\}$ (truncated subtraction). The operators $\oplus$ and $\otimes$ are also known as strong disjunction and conjunction in Łukasiewicz logic [22]. The natural order is $\leq$ (less or equal) on the reals.

Another example is $(\{0,\ldots,k\},\oplus,0,\overline{(\cdot)})$ where $n \oplus m = \min\{n+m,k\}$ and $\overline{n} = k-n$ for $n,m \in \{0,\ldots,k\}$. Both MV-algebras are complete and MV-chains.

Boolean algebras (with disjunction and complement) also form MV-algebras that are complete, but in general not MV-chains.

MV-algebras are the algebraic semantics of Łukasiewicz logic. They can be shown to correspond to intervals of the kind $[0,u]$ in suitable groups, i.e., abelian lattice-ordered groups with a strong unit $u$ [21].

### 3 Non-expansive Functions and Their Approximations

As mentioned in the introduction, our interest is for fixpoints of monotone functions $f : \mathbb{M}^Y \to \mathbb{M}^Y$, where $\mathbb{M}$ is an MV-chain and $Y$ is a finite set. We will see that for non-expansive functions we can over-approximate the sets of points in which a given $a \in \mathbb{M}^Y$ can be increased in a way that is preserved by the application of $f$. This will be the core of the proof rules outlined earlier.

*Non-expansive Functions on MV-Algebras.* For defining non-expansiveness it is convenient to introduce a norm.

**Definition 4 (norm).** *Let $\mathbb{M}$ be an MV-chain and let $Y$ be a finite set. Given $a \in \mathbb{M}^Y$ we define its* norm *as $\|a\| = \max\{a(y) \mid y \in Y\}$.*

Given a finite set $Y$ we extend $\oplus$ and $\otimes$ to $\mathbb{M}^Y$ pointwise. Given $Y' \subseteq Y$ and $\delta \in \mathbb{M}$, we write $\delta_{Y'}$ for the function defined by $\delta_{Y'}(y) = \delta$ if $y \in Y'$ and $\delta_{Y'}(y) = 0$, otherwise. Whenever this does not generate confusion, we write $\delta$ instead of $\delta_Y$. It can be seen that $\|\cdot\|$ has the properties of a norm, i.e., for all $a,b \in \mathbb{M}^Y$ and $\delta \in \mathbb{M}$, it holds that (1) $\|a \oplus b\| \sqsubseteq \|a\| \oplus \|b\|$, (2) $\|\delta \otimes a\| = \delta \otimes \|a\|$ and and $\|a\| = 0$ implies that $a$ is the constant 0. Moreover, it is clearly monotonic, i.e., if $a \sqsubseteq b$ then $\|a\| \sqsubseteq \|b\|$.

We next introduce non-expansiveness. Despite the fact that we will finally be interested in endo-functions $f : \mathbb{M}^Y \to \mathbb{M}^Y$, in order to allow for a compositional reasoning we work with functions where domain and codomain can be different.

**Definition 5 (non-expansiveness).** *Let $f : \mathbb{M}^Y \to \mathbb{M}^Z$ be a function, where $\mathbb{M}$ is an MV-chain and $Y, Z$ are finite sets. We say that it is* non-expansive *if for all $a,b \in \mathbb{M}^Y$ it holds $\|f(b) \ominus f(a)\| \sqsubseteq \|b \ominus a\|$.*

Note that $(a,b) \mapsto \|a \ominus b\|$ is the supremum lifting of a directed version of Chang's distance [21]. It is easy to see that all non-expansive functions on MV-chains are monotone.

*Approximating the Propagation of Increases.* Let $f : \mathbb{M}^Y \to \mathbb{M}^Z$ be a monotone function and take $a,b \in \mathbb{M}^Y$ with $a \sqsubseteq b$. We are interested in the difference $b(y) \ominus a(y)$ for some $y \in Y$ and on how the application of $f$ "propagates" this increase. The reason is that, understanding that no increase can be propagated will be crucial to establish when a fixpoint of a non-expansive function $f$ is actually the largest one, and, more generally, when a (pre-)fixpoint of $f$ is above the largest fixpoint.

In order to formalise the above intuition, we rely on tools from abstract interpretation. In particular, the following pair of functions, which, under a suitable condition, form a Galois connection, will play a major role. The left adjoint $\alpha_{a,\delta}$ takes as input a set $Y'$ and, for $y \in Y'$, it increases the values $a(y)$ by $\delta$, while the right adjoint $\gamma_{a,\delta}$ takes as input a function $b \in \mathbb{M}^Y$, $b \in [a, a \oplus \delta]$ and checks for which parameters $y \in Y$ the value $b(y)$ exceeds $a(y)$ by $\delta$.

We also define $[Y]_a$, the subset of elements in $Y$ where $a(y)$ is not 1 and thus there is a potential to increase, and $\delta_a$, which gives us the minimal such increase.

**Definition 6 (functions to sets, and vice versa).** *Let* $\mathbb{M}$ *be an MV-algebra and let* $Y$ *be a finite set. Define the set* $[Y]_a = \{y \in Y \mid a(y) \neq 1\}$ *and* $\delta_a = \min\{\overline{a(y)} \mid y \in [Y]_a\}$ *with* $\min \emptyset = 1$.

*For* $0 \sqsubset \delta \in \mathbb{M}$ *we consider the functions* $\alpha_{a,\delta} : \mathbf{2}^{[Y]_a} \to [a, a \oplus \delta]$ *and* $\gamma_{a,\delta} : [a, a \oplus \delta] \to \mathbf{2}^{[Y]_a}$, *defined, for* $Y' \in \mathbf{2}^{[Y]_a}$ *and* $b \in [a, a \oplus \delta]$, *by*

$$\alpha_{a,\delta}(Y') = a \oplus \delta_{Y'} \qquad \gamma_{a,\delta}(b) = \{y \in [Y]_a \mid b(y) \ominus a(y) \sqsupseteq \delta\}.$$

When $\delta$ is sufficiently small, the pair $\langle \alpha_{a,\delta}, \gamma_{a,\delta} \rangle$ is a Galois connection.

**Lemma 7 (Galois connection).** *Let* $\mathbb{M}$ *be an MV-algebra and* $Y$ *be a finite set. For* $0 \neq \delta \sqsubseteq \delta_a$, *the pair* $\langle \alpha_{a,\delta}, \gamma_{a,\delta} \rangle : \mathbf{2}^{[Y]_a} \to [a, a \oplus \delta]$ *is a Galois connection.*

$$\mathbf{2}^{[Y]_a} \underset{\gamma_{a,\delta}}{\overset{\alpha_{a,\delta}}{\rightleftarrows}} [a, a \oplus \delta]$$

Whenever $f$ is non-expansive, it is easy to see that it restricts to a function $f : [a, a \oplus \delta] \to [f(a), f(a) \oplus \delta]$ for all $\delta \in \mathbb{M}$.

As mentioned before, a crucial result shows that for all non-expansive functions, under the assumption that $Y, Z$ are finite and the order on $\mathbb{M}$ is total, we can suitably approximate the propagation of increases. In order to state this result, a useful tool is a notion of approximation of a function.

**Definition 8 ($(\delta, a)$-approximation).** *Let* $\mathbb{M}$ *be an MV-chain, let* $Y$, $Z$ *be finite sets and let* $f : \mathbb{M}^Y \to \mathbb{M}^Z$ *be a non-expansive function. For* $a \in \mathbb{M}^Y$ *and any* $\delta \in \mathbb{M}$ *we define* $f^{\#}_{a,\delta} : \mathbf{2}^{[Y]_a} \to \mathbf{2}^{[Z]_{f(a)}}$ *as* $f^{\#}_{a,\delta} = \gamma_{f(a),\delta} \circ f \circ \alpha_{a,\delta}$.

Given $Y' \subseteq [Y]_a$, its image $f^{\#}_{a,\delta}(Y') \subseteq [Z]_{f(a)}$ is the set of points $z \in [Z]_{f(a)}$ such that $\delta \sqsubseteq f(a \oplus \delta_{Y'})(z) \ominus f(a)(z)$, i.e., the points to which $f$ propagates an increase of the function $a$ with value $\delta$ on the subset $Y'$.

We first show that $f^{\#}_{a,\delta}$ is antitone in the parameter $\delta$, a non-trivial result.

**Lemma 9 (anti-monotonicity).** *Let* $\mathbb{M}$ *be an MV-chain, let* $Y$, $Z$ *be finite sets, let* $f : \mathbb{M}^Y \to \mathbb{M}^Z$ *be a non-expansive function and let* $a \in \mathbb{M}^Y$. *For* $\theta, \delta \in \mathbb{M}$, *if* $\theta \sqsubseteq \delta$ *then* $f^{\#}_{a,\delta} \subseteq f^{\#}_{a,\theta}$.

Since $f^{\#}_{a,\delta}$ increases when $\delta$ decreases and there are finitely many such functions, there must be a value $\iota^f_a$ such that all functions $f^{\#}_{a,\delta}$ for $0 \sqsubset \delta \sqsubseteq \iota^f_a$ are equal. This function is denoted by $f^{\#}_a$ and is called the *a-approximation of* $f$.

We next show that indeed, for all non-expansive functions, the $a$-approximation properly approximates the propagation of increases.

**Theorem 10 (approximation of non-expansive functions).** *Let* $\mathbb{M}$ *be a complete MV-chain, let* $Y, Z$ *be finite sets and let* $f : \mathbb{M}^Y \to \mathbb{M}^Z$ *be a non-expansive function. Then there exists* $\iota_a^f \in \mathbb{M}$*, the largest value below or equal to* $\delta_a$ *such that* $f^\#_{a,\delta} = f^\#_{a,\delta'}$ *for all* $0 \sqsubset \delta, \delta' \sqsubseteq \iota_a^f$.

*We denote this function by* $f_a^\#$ *and call it the* $a$-approximation *of* $f$*. Then for all* $0 \sqsubset \delta \in \mathbb{M}$*:*

$$\begin{array}{ccc} [a, a \oplus \delta] & \xrightarrow{\gamma_{a,\delta}} & \mathbf{2}^{[Y]_a} \\ {\scriptstyle f}\downarrow & \sqsubseteq & \downarrow{\scriptstyle f_a^\#} \\ [f(a), f(a) \oplus \delta] & \xrightarrow[\gamma_{f(a),\delta}]{} & \mathbf{2}^{[Z]_{f(a)}} \end{array}$$

*a.* $\gamma_{f(a),\delta} \circ f \subseteq f_a^\# \circ \gamma_{a,\delta}$
*b. for* $\delta \sqsubseteq \delta_a$*:* $\delta \sqsubseteq \iota_a^f$ *iff* $\gamma_{f(a),\delta} \circ f = f_a^\# \circ \gamma_{a,\delta}$

Note that if $Y = Z$ and $a$ is a fixpoint of $f$, i.e., $a = f(a)$, condition (a) above corresponds exactly to soundness in the sense of abstract interpretation [13], while condition (b) corresponds to ($\gamma$-)completeness (see also §2).

### 4 Proof Rules

In this section we formalise the proof technique outlined in the introduction for showing that a fixpoint is the largest and, more generally, for checking over-approximations of greatest fixpoints of non-expansive functions.

Consider a monotone function $f : \mathbb{M}^Y \to \mathbb{M}^Y$ for some finite set $Y$. We first focus on the problem of establishing whether some given fixpoint $a$ of $f$ coincides with $\nu f$ (without explicitly knowing $\nu f$), and, in case it does not, finding an "improvement", i.e., a post-fixpoint of $f$, larger than $a$. Observe that when $a$ is a fixpoint, $[Y]_a = [Y]_{f(a)}$ and thus the $a$-approximation of $f$ (Thm. 10) is an endofunction $f_a^\# : [Y]_a \to [Y]_a$. We have the following result, which relies on the fact that due to Thm. 10 $\gamma_{a,\delta}$ preserves fixpoints (of $f$ and $f_a^\#$).

**Theorem 11 (soundness and completeness for fixpoints).** *Let* $\mathbb{M}$ *be a complete MV-chain,* $Y$ *a finite set and* $f : \mathbb{M}^Y \to \mathbb{M}^Y$ *be a non-expansive function. Let* $a \in \mathbb{M}^Y$ *be a fixpoint of* $f$*. Then* $\nu f_a^\# = \emptyset$ *if and only if* $a = \nu f$.

Whenever $a$ is a fixpoint, but not yet the largest fixpoint of $f$, we can increase it and obtain a post-fixpoint.

**Lemma 12.** *Let* $\mathbb{M}$ *be a complete MV-chain,* $f : \mathbb{M}^Y \to \mathbb{M}^Y$ *a non-expansive function,* $a \in \mathbb{M}$ *a fixpoint of* $f$*, and let* $f_a^\#$ *be the corresponding* $a$*-approximation and* $\iota_a^f$ *as in Thm. 10. Then* $\alpha_{a,\iota_a^f}(\nu f_a^\#) = a \oplus (\iota_a^f)_{\nu f_a^\#}$ *is a post-fixpoint of* $f$.

Using these results one can perform an alternative fixpoint iteration where we iterate to the largest fixpoint from below: start with a post-fixpoint $a_0 \sqsubseteq f(a_0)$ (which is clearly below $\nu f$) and obtain, by (possibly transfinite) iteration, an ascending chain that converges to $a$, the least fixpoint above $a_0$. Now check with Thm. 11 whether $Y' = \nu f_a^\# = \emptyset$. If yes, we have reached $\nu f = a$. If not,

$\alpha_{a,\iota_a^f}(Y') = a \oplus (\iota_a^f)_{Y'}$ is again a post-fixpoint (cf. Lem. 12) and we continue this procedure until – for some ordinal – we reach the largest fixpoint $\nu f$, for which we have $\nu f^{\#}_{\nu f} = \emptyset$.

Interestingly, the soundness result in Thm. 11 can be generalised to the case in which $a$ is a pre-fixpoint instead of a fixpoint. In this case, the $a$-approximation for a function $f : \mathbb{M}^Y \to \mathbb{M}^Y$ is a function $f_a^{\#} : [Y]_a \to [Y]_{f(a)}$ where domain and codomain are different, hence it would not be meaningful to look for fixpoints. However, as explained below, it can be restricted to an endofunction.

**Theorem 13 (soundness for pre-fixpoints).** *Let $\mathbb{M}$ be a complete MV-chain, $Y$ a finite set and $f : \mathbb{M}^Y \to \mathbb{M}^Y$ be a non-expansive function. Given a pre-fixpoint $a \in \mathbb{M}^Y$ of $f$, let $[Y]_{a=f(a)} = \{y \in [Y]_a \mid a(y) = f(a)(y)\}$. Let us define $f_a^* : [Y]_{a=f(a)} \to [Y]_{a=f(a)}$ as $f_a^*(Y') = f_a^{\#}(Y') \cap [Y]_{a=f(a)}$, where $f_a^{\#} : \mathbf{2}^{[Y]_a} \to \mathbf{2}^{[Y]_{f(a)}}$ is the $a$-approximation of $f$. If $\nu f_a^* = \emptyset$ then $\nu f \sqsubseteq a$.*

Roughly, the intuition for the above result is the following: the value of $f(a)$ on some $y$ might or might not depend "circularly" on the value of $a$ on $y$ itself. In a purely inductive setting, without such circular dependencies, $\mu f = \nu f$ and hence $a$ being a pre-fixpoint means that we over-approximate $\nu f$. However, we might have vicious cycles, as explained in the introduction, that destroy the over-approximation since the values are too low. Now, since we restrict to non-expansive functions, it must be the case that there is a cycle, such that all elements on this cycle are points where $a$ and $f(a)$ coincide. It is hence sufficient to check whether a given pre-fixpoint could be increased on its subpart which corresponds to a fixpoint, i.e., the idea is to restrict to $[Y]_{a=f(a)}$. We detect such situations by looking for "wiggle room" as for fixpoints.

Completeness does not generalise to pre-fixpoints, i.e., it is not true that if $a$ is a pre-fixpoint of $f$ and $\nu f \sqsubseteq a$ then $\nu f_a^* = \emptyset$. A pre-fixpoint might contain slack even though it is above the greatest fixpoint. A counterexample is in Ex. 25.

*The Dual View for Least Fixpoints.* The theory developed so far can be easily dualised to check under-approximations of least fixpoints. Given a complete MV-algebra $\mathbb{M} = (M, \oplus, 0, \overline{(\cdot)})$ and a monotone function $f : \mathbb{M}^Y \to \mathbb{M}^Y$, in order to show that a post-fixpoint $a \in \mathbb{M}^Y$ satisfies $a \sqsubseteq \mu f$, we can in fact simply work in the dual MV-algebra, $\mathbb{M}^{op} = (M, \sqsupseteq, \otimes, \overline{(\cdot)}, 1)$. It is convenient to formulate the conditions using $\ominus$ and the original order.

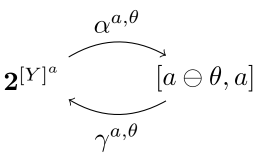

We next outline the dualised setting. The notation for the dual case is obtained from that of the original (primal) case, exchanging subscripts and superscripts.

Given $a \in \mathbb{M}^Y$, define $[Y]^a = \{y \in Y \mid a(y) \neq 0\}$ and $\delta^a = \min\{a(y) \mid y \in [Y]^a\}$. For $\theta \in \mathbb{M}$, we consider the pair of functions $\langle \alpha^{a,\theta}, \gamma^{a,\theta} \rangle : \mathbf{2}^{[Y]^a} \to [a \ominus \theta, a]$ where, for $Y' \in \mathbf{2}^{[Y]^a}$, we let $\alpha^{a,\theta}(Y') = a \ominus \theta_{Y'}$ and, for $b \in [a \ominus \theta, a]$, $\gamma^{a,\theta}(b) = \{y \in Y \mid a(y) \ominus b(y) \sqsupseteq \theta\}$.

A function $f : \mathbb{M}^Y \to \mathbb{M}^Z$ is non-expansive in the dual MV-algebra when it is in the primal one. Its approximation in the sense of Thm. 10 is denoted $f^a_{\#}$.

Table 1: Basic functions $f\colon \mathbb{M}^Y \to \mathbb{M}^Z$ (constant, reindexing, minimum, maximum, average), function composition, disjoint union and the corresponding approximations $f_a^\#\colon \mathbf{2}^{[Y]_a} \to \mathbf{2}^{[Z]_{f(a)}}$, $f_\#^a\colon \mathbf{2}^{[Y]^a} \to \mathbf{2}^{[Z]^{f(a)}}$.

*Notation:* $\mathcal{R}^{-1}(z) = \{y \in Y \mid y\mathcal{R}z\}$, $supp(p) = \{y \in Y \mid p(y) > 0\}$ for $p \in \mathcal{D}(Y)$, $Min_a = \{y \in Y \mid a(y)$ minimal$\}$, $Max_a = \{y \in Y \mid a(y)$ maximal$\}$, $a\colon Y \to \mathbb{M}$

| function $f$ | definition of $f$ | $f_a^\#(Y')$ (above), $f_\#^a(Y')$ (below) |
|---|---|---|
| $c_k$<br>$(k \in \mathbb{M}^Z)$ | $f(a) = k$ | $\emptyset$<br>$\emptyset$ |
| $u^*$<br>$(u\colon Z \to Y)$ | $f(a) = a \circ u$ | $u^{-1}(Y')$<br>$u^{-1}(Y')$ |
| $\min_\mathcal{R}$<br>$(\mathcal{R} \subseteq Y \times Z)$ | $f(a)(z) = \min\limits_{y\mathcal{R}z} a(y)$ | $\{z \in [Z]_{f(a)} \mid Min_{a\vert_{\mathcal{R}^{-1}(z)}} \subseteq Y'\}$<br>$\{z \in [Z]^{f(a)} \mid Min_{a\vert_{\mathcal{R}^{-1}(z)}} \cap Y' \neq \emptyset\}$ |
| $\max_\mathcal{R}$<br>$(\mathcal{R} \subseteq Y \times Z)$ | $f(a)(z) = \max\limits_{y\mathcal{R}z} a(y)$ | $\{z \in [Z]_{f(a)} \mid Max_{a\vert_{\mathcal{R}^{-1}(z)}} \cap Y' \neq \emptyset\}$<br>$\{z \in [Z]^{f(a)} \mid Max_{a\vert_{\mathcal{R}^{-1}(z)}} \subseteq Y'\}$ |
| $\mathrm{av}_D$ $(\mathbb{M} = [0,1]$,<br>$Z = D \subseteq \mathcal{D}(Y))$ | $f(a)(p) = \sum\limits_{y \in Y} p(y) \cdot a(y)$ | $\{p \in [D]_{f(a)} \mid supp(p) \subseteq Y'\}$<br>$\{p \in [D]^{f(a)} \mid supp(p) \subseteq Y'\}$ |
| $h \circ g$<br>$(g\colon \mathbb{M}^Y \to \mathbb{M}^W$,<br>$h\colon \mathbb{M}^W \to \mathbb{M}^Z)$ | $f(a) = h(g(a))$ | $h_{g(a)}^\# \circ g_a^\#(Y')$<br>$h_\#^{g(a)} \circ g_\#^a(Y')$ |
| $\biguplus\limits_{i \in I} f_i$ $I$ finite<br>$(f_i\colon \mathbb{M}^{Y_i} \to \mathbb{M}^{Z_i}$,<br>$Y = \bigcup\limits_{i \in I} Y_i$, $Z = \biguplus\limits_{i \in I} Z_i)$ | $f(a)(z) = f_i(a\vert_{Y_i})(z)$<br>$(z \in Z_i)$ | $\biguplus_{i \in I} (f_i)_{a\vert_{Y_i}}^\#(Y' \cap Y_i)$<br>$\biguplus_{i \in I} (f_i)_\#^{a\vert_{Y_i}}(Y' \cap Y_i)$ |

Then the dualisations of Thm. 11 and 13 hold, i.e., if $a$ is a fixpoint of $f$, then $\nu f_\#^a = \emptyset$ iff $\mu f = a$, and whenever $a$ is a post-fixpoint, $\nu f_*^a = \emptyset$ implies $a \sqsubseteq \mu f$.

### 5 (De)Composing Functions and Approximations

Given a non-expansive function $f$ and a (pre/post-)fixpoint $a$, it is often non-trivial to determine the corresponding approximations. However, non-expansive functions enjoy good closure properties (closure under composition, and closure under disjoint union) and we will see that the same holds for the corresponding approximations. Furthermore it turns out that the functions needed in the applications can be obtained from just a few templates. This gives us a toolbox for assembling approximations with relative ease.

**Theorem 14.** *All basic functions listed in Table 1 are non-expansive. Furthermore non-expansive functions are closed under composition and disjoint union. The approximations are the ones listed in the third column of the table.*

## 6 Applications

### 6.1 Termination Probability

We start by making the example from the introduction (§1) more formal. Consider a Markov chain $(S, T, \eta)$, as defined in the introduction (Fig. 1), where we restrict the codomain of $\eta\colon S\backslash T \to \mathcal{D}(S)$ to $D \subseteq \mathcal{D}(S)$, where $D$ is finite (to ensure that all involved sets are finite). Furthermore let $\mathcal{T}\colon [0,1]^S \to [0,1]^S$ be the function from the introduction whose least fixpoint $\mu\mathcal{T}$ assigns to each state its termination probability.

**Lemma 15.** *The function $\mathcal{T}$ can be written as $\mathcal{T} = (\eta^* \circ \mathrm{av}_D) \uplus c_k$ where $k\colon T \to [0,1]$ is the constant function 1 defined only on terminal states.*

From this representation and Thm. 14 it is obvious that $\mathcal{T}$ is non-expansive.

**Lemma 16.** *Let $t\colon S \to [0,1]$. The approximation for $\mathcal{T}$ in the dual sense is $\mathcal{T}_\#^t\colon \mathbf{2}^{[S]^t} \to \mathbf{2}^{[S]^{\mathcal{T}(t)}}$ with*

$$\mathcal{T}_\#^t(S') = \{s \in [S]^{\mathcal{T}(t)} \mid s \notin T \wedge \mathit{supp}(\eta(s)) \subseteq S'\}.$$

It is well-known that the function $\mathcal{T}$ can be tweaked in such a way that it has a unique fixpoint, coinciding with $\mu\mathcal{T}$, by determining all states which cannot reach a terminal state and setting their value to zero [3]. Hence fixpoint iteration from above does not bring us any added value here. It does however make sense to use the proof rule in order to guarantee lower bounds via post-fixpoints.

Furthermore, termination probability is a special case of the considerably more complex stochastic games that will be studied in §7, where the trick of modifying the function is not applicable.

### 6.2 Behavioural Metrics for Probabilistic Automata

Before we start discussing probabilistic automata, we first consider the Hausdorff and the Kantorovich lifting and the corresponding approximations.

*Hausdorff Lifting.* Given a metric on a set $X$, the Hausdorff metric is obtained by lifting the original metric to $\mathbf{2}^X$. Here we define this for general distance functions on $\mathbb{M}$, not restricting to metrics. In particular the Hausdorff lifting is given by a function $\mathcal{H} : \mathbb{M}^{X\times X} \to \mathbb{M}^{\mathbf{2}^X \times \mathbf{2}^X}$ where

$$\mathcal{H}(d)(X_1, X_2) = \max\{\max_{x_1\in X_1}\min_{x_2\in X_2} d(x_1,x_2), \max_{x_2\in X_2}\min_{x_1\in X_1} d(x_1,x_2)\}.$$

An alternative characterisation due to Mémoli [20], also in [4], is more convenient for our purposes. If we let $u : \mathbf{2}^{X\times X} \to \mathbf{2}^X \times \mathbf{2}^X$ with $u(C) = (\pi_1[C], \pi_2[C])$, where $\pi_1, \pi_2$ are the projections $\pi_i : X \times X \to X$ and $\pi_i[C] = \{\pi_i(c) \mid c \in C\}$. Then $\mathcal{H}(d)(X_1, X_2) = \min\{\max_{(x_1,x_2)\in C} d(x_1,x_2) \mid C \subseteq X \times X \ \wedge\ u(C) = (X_1, X_2)\}$. Relying on this, we can obtain the result below, from which we deduce that $\mathcal{H}$ is non-expansive and construct its approximation as the composition of the corresponding functions from Table 1.

**Lemma 17.** $\mathcal{H} = \min_u \circ \max_\in$ *where* $\max_\in : \mathbb{M}^{X\times X} \to \mathbb{M}^{\mathbf{2}^{X\times X}}$ *($\in\, \subseteq (X\times X)\times \mathbf{2}^{X\times X}$ is the "is-element-of"-relation on $X \times X$),* $\min_u : \mathbb{M}^{\mathbf{2}^{X\times X}} \to \mathbb{M}^{\mathbf{2}^X\times\mathbf{2}^X}$.

*Kantorovich Lifting.* The Kantorovich (also known as Wasserstein) lifting converts a metric on $X$ to a metric on probability distributions over $X$. As for the Hausdorff lifting, we lift distance functions that are not necessarily metrics.

Furthermore, in order to ensure finiteness of all the sets involved, we restrict to $D \subseteq \mathcal{D}(X)$, some finite set of probability distributions over $X$. A *coupling* of $p, q \in D$ is a probability distribution $c \in \mathcal{D}(X \times X)$ whose left and right marginals are $p, q$, i.e., $p(x_1) = m_c^L(x_1) := \sum_{x_2\in X} c(x_1, x_2)$ and $q(x_2) = m_c^R(x_2) := \sum_{x_1\in X} c(x_1, x_2)$. The set of all couplings of $p, q$, denoted by $\Omega(p, q)$, forms a polytope with finitely many vertices [24]. The set of all polytope vertices that are obtained by coupling any $p, q \in D$ is also finite and is denoted by $VP_D \subseteq \mathcal{D}(X \times X)$.

The Kantorovich lifting is given by $\mathcal{K} : [0,1]^{X\times X} \to [0,1]^{D\times D}$ where

$$\mathcal{K}(d)(p,q) = \min_{c\in\Omega(p,q)} \sum_{(x_1,x_2)\in X\times X} c(x_1,x_2)\cdot d(x_1,x_2).$$

The coupling $c$ can be interpreted as the optimal transport plan to move goods from suppliers to customers [30]. Again there is an alternative characterisation, which shows non-expansiveness of $\mathcal{K}$:

**Lemma 18.** *Let* $u : VP_D \to D\times D$, $u(c) = (m_c^L, m_c^R)$*. Then* $\mathcal{K} = \min_u \circ \mathrm{av}_{VP_D}$*, where* $\mathrm{av}_{VP_D} : [0,1]^{X\times X} \to [0,1]^{VP_D}$*,* $\min_u : [0,1]^{VP_D} \to [0,1]^{D\times D}$*.*

*Probabilistic Automata.* We now compare our approach with [2], which describes the first method for computing behavioural distances for probabilistic automata. Although the behavioural distance arises as a least fixpoint, it is in fact better, even the only known method, to iterate from above, in order to reach this least fixpoint. This is done by guessing and improving couplings, similar to strategy iteration discussed later in §7. A major complication, faced in [2], is that the procedure can get stuck at a fixpoint which is not the least and one has to determine that this is the case and decrease the current candidate. In fact this paper was our inspiration to generalise this technique to a more general setting.

A *probabilistic automaton* is a tuple $\mathcal{A} = (S, L, \eta, \ell)$, where $S$ is a non-empty finite set of states, $L$ is a finite set of labels, $\eta : S \to \mathbf{2}^{\mathcal{D}(S)}$ assigns finite sets of probability distributions to states and $\ell : S \to L$ is a labelling function. (In the following we again replace $\mathcal{D}(S)$ by a finite subset $D$.)

The *probabilistic bisimilarity pseudometrics* is the least fixpoint of the function $\mathcal{M} : [0,1]^{S\times S} \to [0,1]^{S\times S}$ where for $d : S \times S \to [0,1]$, $s, t \in S$:

$$\mathcal{M}(d)(s,t) = \begin{cases} 1 & \text{if } \ell(s) \neq \ell(t) \\ \mathcal{H}(\mathcal{K}(d))(\eta(s), \eta(t)) & \text{otherwise} \end{cases}$$

where $\mathcal{H}$ is the Hausdorff lifting (for $\mathbb{M} = [0,1]$) and $\mathcal{K}$ is the Kantorovich lifting defined earlier. Now assume that $d$ is a fixpoint of $\mathcal{M}$, i.e., $d = \mathcal{M}(d)$. In order to check whether $d = \mu f$, [2] adapts the notion of a self-closed relation from [16].

**Definition 19 ([2]).** *A relation $M \subseteq S \times S$ is* self-closed *wrt. $d = \mathcal{M}(d)$ if, whenever $s\,M\,t$, then*

- $\ell(s) = \ell(t)$ *and* $d(s,t) > 0$,
- *if* $p \in \eta(s)$ *and* $d(s,t) = \min_{q' \in \eta(t)} \mathcal{K}(d)(p,q')$, *then there exists* $q \in \eta(t)$ *and* $c \in \Omega(p,q)$ *such that* $d(s,t) = \sum_{u,v \in S} d(u,v) \cdot c(u,v)$ *and* $supp(c) \subseteq M$,
- *if* $q \in \eta(t)$ *and* $d(s,t) = \min_{p' \in \eta(s)} \mathcal{K}(d)(p',q)$, *then there exists* $p \in \eta(s)$ *and* $c \in \Omega(p,q)$ *such that* $d(s,t) = \sum_{u,v \in S} d(u,v) \cdot c(u,v)$ *and* $supp(c) \subseteq M$.

The largest self-closed relation, denoted by $\approx_d$ is empty if and only if $d = \mu f$ [2]. We now investigate the relation between self-closed relations and post-fixpoints of approximations. For this we will first show that $\mathcal{M}$ can be composed from non-expansive functions, which proves that it is indeed non-expansive. Furthermore, this decomposition will help in the comparison.

**Lemma 20.** *The fixpoint function $\mathcal{M}$ characterizing probabilistic bisimilarity pseudometrics can be written as:*

$$\mathcal{M} = \max_\rho \circ (((\eta \times \eta)^* \circ \mathcal{H} \circ \mathcal{K}) \uplus c_l)$$

*where $\rho\colon (S \times S) \uplus (S \times S) \to (S \times S)$ with $\rho((s,t),i) = (s,t)$.*[4] *Furthermore $l\colon S \times S \to [0,1]$ is defined as $l(s,t) = 0$ if $\ell(s) = \ell(t)$ and $l(s,t) = 1$ if $\ell(s) \neq \ell(t)$.*

Hence $\mathcal{M}$ is a composition of non-expansive functions and thus non-expansive itself. We do not spell out $\mathcal{M}^d_\#$ explicitly, but instead show how it is related to self-closed relations.

**Proposition 21.** *Let $d\colon S \times S \to [0,1]$ where $d = \mathcal{M}(d)$. Then $\mathcal{M}^d_\# \colon \mathbf{2}^{[S\times S]^d} \to \mathbf{2}^{[S\times S]^d}$, where $[S \times S]^d = \{(s,t) \in S \times S \mid d(s,t) > 0\}$.*

*Then $M$ is a self-closed relation wrt. $d$ if and only if $M \subseteq [S \times S]^d$ and $M$ is a post-fixpoint of $\mathcal{M}^d_\#$.*

#### 6.3 Bisimilarity

In order to define standard bisimilarity we use a variant $\mathcal{G}$ of the Hausdorff lifting $\mathcal{H}$ from §6.2 where max and min are swapped and which we denote by $\mathcal{G}$.

Now we can define the fixpoint function for bisimilarity and its corresponding approximation. For simplicity we consider unlabelled transition systems, but it would be straightforward to handle labelled transitions.

Let $X$ be a finite set of states and $\eta : X \to \mathbf{2}^X$ a function that assigns a set of successors $\eta(x)$ to a state $x \in X$. For the fixpoint function for bisimilarity $\mathcal{B} : \{0,1\}^{X\times X} \to \{0,1\}^{X\times X}$ we use the Hausdorff lifting $\mathcal{G}$ with $\mathbb{M} = \{0,1\}$.

**Lemma 22.** *Bisimilarity on $\eta$ is the greatest fixpoint of $\mathcal{B} = (\eta \times \eta)^* \circ \mathcal{G}$.*

[4] Here we use $i \in \{0,1\}$ as indices to distinguish the elements in the disjoint union.

Since we are interested in the greatest fixpoint, we are working in the primal sense. Bisimulation relations are represented by their characteristic functions $d\colon X \times X \to \{0,1\}$, in fact the corresponding relation can be obtained by taking the complement of $[X \times X]_d = \{(x_1, x_2) \in X_1 \times X_2 \mid d(x_1, x_2) = 0\}$.

**Lemma 23.** *Let $d\colon X \times X \to \{0,1\}$. The approximation for the bisimilarity function $\mathcal{B}$ in the primal sense is $\mathcal{B}_d^{\#}\colon \mathbf{2}^{[X\times X]_d} \to \mathbf{2}^{[X\times X]_{\mathcal{B}(d)}}$ with*

$$\begin{aligned}\mathcal{B}_d^{\#}(R) = \{(x_1,x_2) \in [X \times X]_{\mathcal{B}(d)} \mid \\ \forall y_1 \in \eta(x_1) \exists y_2 \in \eta(x_2)\big((y_1,y_2) \notin [X \times X]_d \vee (y_1,y_2) \in R\big) \\ \wedge \forall y_2 \in \eta(x_2) \exists y_1 \in \eta(x_1)\big((y_1,y_2) \notin [X \times X]_d \vee (y_1,y_2) \in R\big)\}\end{aligned}$$

We conclude this section by discussing how this view on bisimilarity can be useful: first, it again opens up the possibility to compute bisimilarity – a greatest fixpoint – by iterating from below, through smaller fixpoints. This could potentially be useful if it is easy to compute the least fixpoint of $\mathcal{B}$ inductively and continue from there.

Furthermore, we obtain a technique for witnessing non-bisimilarity of states. While this can also be done by exhibiting a distinguishing modal formula [17,9] or by a winning strategy for the spoiler in the bisimulation game [27], to our knowledge there is no known method that does this directly, based on the definition of bisimilarity.

With our technique however, we can witness non-bisimilarity of two states $x_1, x_2 \in X$ by presenting a pre-fixpoint $d$ (i.e., $\mathcal{B}(d) \le d$) such that $d(x_1, x_2) = 0$ (equivalent to $(x_1, x_2) \in [X \times X]_d$) and $\nu\mathcal{B}_d^{\#} = \emptyset$, since this implies $\nu\mathcal{B}(x_1, x_2) \le d(x_1, x_2) = 0$ by our proof rule.

There are two issues to discuss: first, how can we characterise a pre-fixpoint of $\mathcal{B}$ (which is quite unusual, since bisimulations are post-fixpoints)? In fact, the condition $\mathcal{B}(d) \le d$ can be rewritten to: for all $(x_1, x_2) \in [X \times X]_d$ there exists $y_1 \in \eta(x_1)$ such that for all $y_2 \in \eta(x_2)$ we have $(y_1, y_2) \in [X \times X]_d$ (*or* vice versa). Second, at first sight it does not seem as if we gained anything since we still have to do a fixpoint computation on relations. However, the carrier set is $[X \times X]_d$, i.e., a set of non-bisimilarity witnesses and this set can be small even though $X$ might be large.

*Example 24.* We consider the transition system depicted below.

Our aim is to construct a witness showing that $x, u$ are not bisimilar. This witness is a function $d\colon X \times X \to \{0,1\}$ with $d(x,u) = 0 = d(y,u)$ and for all other pairs the value is 1.

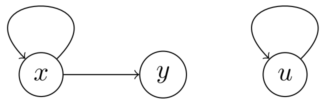

Hence $[X \times X]_{d=\mathcal{B}(d)} = [X \times X]_d = \{(x,u),(y,u)\}$ and it is easy to check that $d$ is a pre-fixpoint of $\mathcal{B}$ and that $\nu\mathcal{B}_d^* = \emptyset$: we iterate over $\{(x,u),(y,u)\}$ and first remove $(y,u)$ (since $y$ has no successors) and then $(x,u)$. This implies that $\nu\mathcal{B} \le d$ and hence $\nu\mathcal{B}(x,u) = 0$, which means that $x, u$ are not bisimilar.

*Example 25.* We modify Ex. 24 and consider a function $d$ where $d(x,u) = 0$ and all other values are 1. Again $d$ is a pre-fixpoint of $\mathcal{B}$ and $\nu\mathcal{B} \leq d$ (since only reflexive pairs are in the bisimilarity). However $\nu\mathcal{B}_d^* \neq \emptyset$, since $\{(x,u)\}$ is a post-fixpoint. This is a counterexample to completeness discussed after Thm. 13.

Intuively speaking, the states $y, u$ over-approximate and claim that they are bisimilar, although they are not. (This is permissible for a pre-fixpoint.) This tricks $x, u$ into thinking that there is some wiggle room and that one can increase the value of $(x,u)$. This is true, but only because of the limited, local view, since the "true" value of $(y,u)$ is 0.

### 7 Simple Stochastic Games

*Introduction to Simple Stochastic Games.* In this section we show how our techniques can be applied to simple stochastic games [11,10]. A simple stochastic game is a state-based two-player game where the two players, Min and Max, each own a subset of states they control, for which they can choose the successor. The system also contains sink states with an assigned payoff and averaging states which randomly choose their successor based on a given probability distribution. The goal of Min is to minimise and the goal of Max to maximise the payoff.

Simple stochastic games are an important type of games that subsume parity games and the computation of behavioural distances for probabilistic automata (cf. §6.2, [2]). The associated decision problem is known to lie in $\mathsf{NP} \cap \mathsf{coNP}$, but it is an open question whether it is contained in $\mathsf{P}$. There are known randomised subexponential algorithms [7].

It has been shown that it is sufficient to consider positional strategies, i.e., strategies where the choice of the player is only dependent on the current state. The expected payoffs for each state form a so-called value vector and can be obtained as the least solution of a fixpoint equation (see below).

A *simple stochastic game* is given by a finite set $V$ of nodes, partitioned into $\mathit{MIN}$, $\mathit{MAX}$, $\mathit{AV}$ (average) and $\mathit{SINK}$, and the following data: $\eta_{\min} : \mathit{MIN} \to \mathbf{2}^V$, $\eta_{\max} : \mathit{MAX} \to \mathbf{2}^V$ (successor functions for Min and Max nodes), $\eta_{\mathrm{av}} : \mathit{AV} \to D$ (probability distributions, where $D \subseteq \mathcal{D}(V)$ finite) and $w : \mathit{SINK} \to [0,1]$ (weights of sink nodes).

The fixpoint function $\mathcal{V}\colon [0,1]^V \to [0,1]^V$ is defined below for $a\colon V \to [0,1]$ and $v \in V$:

$$\mathcal{V}(a)(v) = \begin{cases} \min_{v' \in \eta_{\min}(v)} a(v') & v \in \mathit{MIN} \\ \max_{v' \in \eta_{\max}(v)} a(v') & v \in \mathit{MAX} \\ \sum_{v' \in V} \eta_{\mathrm{av}}(v)(v') \cdot a(v') & v \in \mathit{AV} \\ w(v) & v \in \mathit{SINK} \end{cases}$$

The *least* fixpoint of $\mathcal{V}$ specifies the average payoff for all nodes when Min and Max play optimally. In an infinite game the payoff is 0. In order to avoid infinite games and guarantee uniqueness of the fixpoint, many authors [18,10,29] restrict to stopping games, which are guaranteed to terminate for every pair of Min/Max-strategies. Here we deal with general games where more than one fixpoint may exist. Such a scenario has been studied in [19], which considers value iteration to under- and over-approximate the value vector. The over-approximation faces challenges with cyclic dependencies, similar to the vicious cycles described earlier. Here we focus on strategy iteration, which is usually less efficient than value iteration, but yields a precise result instead of approximating it.

*Example 26.* We consider the game depicted below. Here min is a Min node with $\eta_{\mathrm{min}}(\mathrm{min}) = \{\mathbf{1}, \mathrm{av}\}$, max is a Max node with $\eta_{\mathrm{max}}(\mathrm{max}) = \{\boldsymbol{\varepsilon}, \mathrm{av}\}$, $\mathbf{1}$ is a sink node with payoff 1, $\boldsymbol{\varepsilon}$ is a sink node with some small payoff $\varepsilon \in (0,1)$ and av is an average node which transitions to both min and max with probability $\frac{1}{2}$.

Min should choose av as successor since a payoff of 1 is bad for Min. Given this choice of Min, Max should not declare av as successor since this would create an infinite play and hence the payoff is 0. Therefore Max has to choose $\boldsymbol{\varepsilon}$ and be content with a payoff of $\varepsilon$, which is achieved from all nodes different from $\mathbf{1}$.

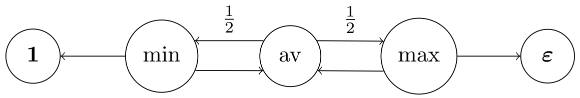

In order to be able to determine the approximation of $\mathcal{V}$ and to apply our techniques, we consider the following equivalent definition.

**Lemma 27.** *$\mathcal{V} = (\eta^*_{\mathrm{min}} \circ \mathrm{min}_\in) \uplus (\eta^*_{\mathrm{max}} \circ \mathrm{max}_\in) \uplus (\eta^*_{\mathrm{av}} \circ \mathrm{av}_D) \uplus c_w$, where $\in \subseteq V \times \mathbf{2}^V$ is the "is-element-of"-relation on $V$.*

As a composition of non-expansive functions, $\mathcal{V}$ is non-expansive as well. Since we are interested in the least fixpoint we work in the dual sense and obtain the following approximation, which intuitively says: we can decrease a value at node $v$ by a constant only if, in the case of a Min node, we decrease the value of one successor where the minimum is reached, in the case of a Max node, we decrease the values of all successors where the maximum is reached, and in the case of an average node, we decrease the values of all successors.

**Lemma 28.** *Let $a \colon V \to [0,1]$. The approximation for the value iteration function $\mathcal{V}$ in the dual sense is $\mathcal{V}^a_\#\colon \mathbf{2}^{[V]^a} \to \mathbf{2}^{[V]^{\mathcal{V}(a)}}$ with*

$$\mathcal{V}^a_\#(V') = \{v \in [V]^{\mathcal{V}(a)} \mid \big(v \in \mathit{MIN} \wedge \mathit{Min}_{a|_{\eta_{\mathrm{min}}(v)}} \cap V' \neq \emptyset\big) \vee \big(v \in \mathit{MAX} \wedge \mathit{Max}_{a|_{\eta_{\mathrm{max}}(v)}} \subseteq V'\big) \vee \big(v \in \mathit{AV} \wedge \mathit{supp}(\eta_{\mathrm{av}}(v)) \subseteq V'\big)\}$$

*Strategy Iteration from Above and Below.* We describe two algorithms based on strategy iteration, first introduced by Hoffman and Karp in [18], that are novel, as far as we know. The first iterates to the least fixpoint from above and uses the techniques described in §4. The second iterates from below: the role of our results is not directly visible in the code of the algorithm, but its non-trivial correctness proof is based on the proof rule introduced earlier.

**Determine $\mu\mathcal{V}$ (from above)**

1. Guess a Min-strategy $\tau^{(0)}$, $i := 0$
2. $a^{(i)} := \mu\mathcal{V}_{\tau^{(i)}}$
3. $\tau^{(i+1)} := sw_{\min}(\tau^{(i)}, a^{(i)})$
4. If $\tau^{(i+1)} \neq \tau^{(i)}$ $i := i + 1$ then goto 2.
5. Compute $V' = \nu\mathcal{V}^a_\#$, where $a = a^{(i)}$.
6. If $V' = \emptyset$ then stop and return $a^{(i)}$. Otherwise set $a^{(i+1)} := a - (\iota^a_\mathcal{V})_{V'}$, $\tau^{(i+2)} := sw_{\min}(\tau^{(i)}, a^{(i+1)})$, $i := i+2$, goto 2.

(a) Strategy iteration from above

**Determine $\mu\mathcal{V}$ (from below)**

1. Guess a Max-strategy $\sigma^{(0)}$, $i := 0$
2. $a^{(i)} := \mu\mathcal{V}_{\sigma^{(i)}}$
3. $\sigma^{(i+1)} := sw_{\max}(\sigma^{(i)}, a^{(i)})$
4. If $\sigma^{(i+1)} \neq \sigma^{(i)}$ set $i := i+1$ and goto 2. Otherwise stop and return $a^{(i)}$.

(b) Strategy iteration from below

Fig. 2: Strategy iteration from above and below

We first recap the underlying notions: a Min-strategy is a mapping $\tau\colon \mathit{MIN} \to V$ such that $\tau(v) \in \eta_{\min}(v)$ for every $v \in \mathit{MIN}$. With such a strategy, Min decides to always leave a node $v$ via $\tau(v)$. Analogously $\sigma\colon \mathit{MAX} \to V$ fixes a Max-strategy. Fixing a strategy for either player induces a modified value function. If $\tau$ is a Min-strategy, we obtain $\mathcal{V}_\tau$ which is defined exactly as $\mathcal{V}$ but for $v \in \mathit{MIN}$ where we set $\mathcal{V}_\tau(a)(v) = a(\tau(v))$. Analogously, for $\sigma$ a Max-strategy, $\mathcal{V}_\sigma$ is obtained by setting $\mathcal{V}_\sigma(a)(v) = a(\sigma(v))$ when $v \in \mathit{MAX}$. If both players fix their strategies, the game reduces to a Markov chain.

In order to describe our algorithms we also need the notion of a *switch*. Assume that $\tau$ is a Min-strategy and let $a$ be a (pre-)fixpoint of $\mathcal{V}_\tau$. Min can now potentially improve her strategy for nodes $v \in \mathit{MIN}$ where $\min_{v' \in \eta_{\min}(v)} a(v') < a(\tau(v))$, called *switch nodes*. This results in a Min-strategy $\tau' = sw_{\min}(\tau, a)$, where[5] $\tau'(v) = \arg\min_{v' \in \eta_{\min}(v)} a^{(i)}(v')$ for a switch node $v$ and $\tau'$, $\tau$ agree otherwise. Also, $sw_{\max}(\sigma, a)$ is defined analogously for Max strategies.

Now strategy iteration from above works as described in Figure 2a. The computation of $\mu\mathcal{V}_{\tau^{(i)}}$ in the second step intuitively means that Max chooses his best answering strategy and we compute the least fixpoint based on this answering strategy. At some point no further switches are possible and we have reached a fixpoint $a$, which need not yet be the least fixpoint. Hence we use the techniques from §4 to decrease $a$ and obtain a new pre-fixpoint $a^{(i+1)}$, from which we can continue. The correctness of this procedure partially follows from Thm. 11 and Lem. 12, however we also need to show the following: first, we can compute $a^{(i)} = \mu\mathcal{V}_{\tau^{(i)}}$ efficiently by solving a linear program (cf. Lem. 29) by adapting [11]. Second, the chain of the $a^{(i)}$ decreases, which means that the algorithm will eventually terminate (cf. Thm. 30).

[5] If the minimum is achieved in several nodes, Min simply chooses one of them. However, she will only switch if this strictly improves the value.

Strategy iteration from below is given in Figure 2b. At first sight, the algorithm looks simpler than strategy iteration from above, since we do not have to check whether we have already reached $\nu\mathcal{V}$, reduce and continue from there. However, in this case the computation of $\mu\mathcal{V}_{\sigma^{(i)}}$ via a linear program is more involved (cf. Lem. 29), since we have to pre-compute (via greatest fixpoint iteration over $\mathbf{2}^V$) the nodes where Min can force a cycle based on the current strategy of Max, thus obtaining payoff 0.

This algorithm does not directly use our technique but we can use our proof rules to prove the correctness of the algorithm (Thm. 30). In particular, the proof that the sequence $a^{(i)}$ increases is quite involved: we have to show that $a^{(i)} = \mu\mathcal{V}_{\sigma^{(i)}} \leq \mu\mathcal{V}_{\sigma^{(i+1)}} = a^{(i+1)}$. We prove this, using our proof rules, by showing that $a^{(i)}$ is below the least fixpoint of $\mathcal{V}_{\sigma^{(i+1)}}$.

The algorithm generalises strategy iteration by Hoffman and Karp [18]. Note that we cannot simply adapt their proof, since we do not assume that the game is stopping, which is a crucial ingredient.

**Lemma 29.** *The least fixpoints of $\mathcal{V}_\tau$ and $\mathcal{V}_\sigma$ can be determined by solving linear programs.*

**Theorem 30.** *Strategy iteration from above and below both terminate and compute the least fixpoint of $\mathcal{V}$.*

*Example 31.* Ex. 26 is well suited to explain our two algorithms.

Starting with strategy iteration from above, we may guess $\tau^{(0)}(\text{min}) = \mathbf{1}$. In this case, Max would choose av as successor and we would reach a fixpoint, where each node except for $\varepsilon$ is associated with a payoff of 1. Next, our algorithm would detect the vicious cycle formed by min, av and max. We can reduce the values in this vicious cycle and reach the correct payoff values for each node.

For strategy iteration from below assume that $\sigma^{(0)}(\text{max}) = \text{av}$. Given this strategy of Max, Min can force the play to stay in a cycle formed by min, av and max. Thus, the payoff achieved by the Max strategy $\sigma^{(0)}$ and an optimal play by Min would be 0 for each of these nodes. In the next iteration Max switches and chooses $\varepsilon$ as successor, i.e. $\sigma^{(1)}(\text{max}) = \varepsilon$, which results in the correct values.

We implemented strategy iteration from above and below and classical Kleene iteration in MATLAB. In Kleene iteration we terminate with a tolerance of $10^{-14}$, i.e., we stop if the change from one iteration to the next is below this bound. We tested the algorithms on random stochastic games and found that Kleene iteration is always the fastest, but only converges and it is known that the rate of convergence can be exponentially slow [10]. Strategy iteration from below is usually slightly faster than strategy iteration from above. More details can be found in the full version [5].

### 8 Conclusion

It is well-known that several computations in the context of system verification can be performed by various forms of fixpoint iteration and it is worthwhile to study such methods at a high level of abstraction, typically in the setting of complete lattices and monotone functions. Going beyond the classical results by Tarski [28], combination of fixpoint iteration with approximations [14,6] and with up-to techniques [25] has proven to be successful. Here we treated a more specific setting, where the carrier set consists of functions from a finite set into an MV-chain and the fixpoint functions are non-expansive (and hence monotone), and introduced a novel technique to obtain upper bounds for greatest and lower bounds for least fixpoints, including associated algorithms. Such techniques are widely applicable to a wide range of examples and so far they have been studied only in quite specific scenarios, such as in [2,16,19].

In the future we plan to lift some of the restrictions of our approach. First, an extension to an infinite domain $Y$ would of course be desirable, but since several of our results currently depend on finiteness, such a generalisation does not seem to be easy. Another restriction, to total orders, seems easier to lift: in particular, if the partially ordered MV-algebra $\bar{\mathbb{M}}$ is of the form $\mathbb{M}^I$ where $I$ is a finite index set and $\mathbb{M}$ an MV-chain. (E.g., finite Boolean algebras are of this type.) Then our function space is $\bar{\mathbb{M}}^Y = \left(\mathbb{M}^I\right)^Y \cong \mathbb{M}^{Y\times I}$ and we have reduced to the setting presented in this paper. This will allow us to handle featured transition systems [12] where transitions are equipped with boolean formulas. We also plan to determine the largest possible increase that can be added to a fixpoint that is not yet the greatest fixpoint in order to maximally speed up fixpoint iteration from below (this might be larger than $\iota_a^f$).

There are several other application examples that did not fit into this paper, but that can also be handled by our approach: for instance behavioural distances for metric transition systems [15] and other types of systems [4]. We also plan to investigate other types of games, such as energy games [8]. While here we introduced strategy iteration techniques for simple stochastic games, we also want to check whether we can provide an improvement to value iteration techniques, combining our approach with [19].

We also plan to study whether some examples can be handled with other types of Galois connections: here we used an additive variant, but looking at multiplicative variants (multiplication by a constant factor) might also be fruitful.

*Acknowledgements:* We are grateful to Ichiro Hasuo for making us aware of stochastic games as application domain. Furthermore we would like to thank Matthias Kuntz and Timo Matt for their help with experiments.
